# Complete genome sequences of two strains of *Pseudomonas fragi*, isolated from feces of a domestic rabbit (*Oryctolagus cuniculus*)

**DOI:** 10.1128/mra.00984-25

**Published:** 2025-10-16

**Authors:** L. K. Y. Chow, S. H. N. Pang, K. M. Leung, G. K. K. Lai, S. D. J. Griffin

**Affiliations:** 1 Shuyuan Molecular Biology Laboratory, The Independent Schools Foundation Academy, Hong Kong SAR, China; Rochester Institute of Technology, Rochester, New York, USA

**Keywords:** *Pseudomonas fragi*, malachite green, dye degradation, bioremediation

## Abstract

The complete genomes of *Pseudomonas fragi* strains LKYC.ZH and LKYC.Zb1, recovered from rabbit feces, were established by hybrid assembly: each comprises a single circular chromosome and circular plasmid, totaling 5,036,311 bp (59.06% G+C) and 5,249,147 bp (59.31% G+C), respectively.

## ANNOUNCEMENT

As a psychrotrophic member of the *Pseudomonas fluorescens* group, *Pseudomonas fragi* is commonly known for the aerobic spoilage of chilled foods, such as milk and meat (1–4). However, strains may also be plant growth-promoting (5–8), antifungal (9), nematicidal (7), and algicidal (10). Its versatile biochemistry has been examined for the synthesis of feedstocks and nanomaterials (8, 11), and the species shows potential for bioremediation, with whole cells and enzymes showing useful activity even at low temperatures (11–14). Here, *P. fragi* strains LKYC.ZH and LKYC.Zb1, recovered from feces of a domestic rabbit (*Oryctolagus cuniculus),* were found to degrade the triarylmethane dye malachite green over a broad temperature range.

A sample of rabbit feces (1 g; from 22.3759876° N, 114.1058617° E) was vortexed in 19-mL saline (0.9% wt/vol) and, following a further 10,000-fold dilution, a 100-µL aliquot was spread onto Luria agar (15). Following 24 h of incubation, colonies were transferred to Luria agar containing 0.1 g/L malachite green. Colonies decolorizing the dye were streaked >5 times on Luria agar to generate pure isolates and their decolorizing activity confirmed at temperatures between 4°C and 37°C by inoculating Luria broth containing 20 mg/L malachite green and following a decrease in absorbance due to the dye at 619 nm. For subsequent DNA extraction, a single colony of each pure isolate was streaked onto Luria agar to harvest overnight growth from the plate for processing using the Qiagen DNeasy PowerSoil Pro Kit following the manufacturer’s protocol. All agar incubations were at 27°C.

UV absorbance (BioDrop µLITE; Biochrom Ltd., UK) and PicoGreen assays (16) were used for DNA quality/quantity evaluations before preparing paired-end short-read sequencing libraries (NEBNext Ultra DNA Library Prep Kit) for sequencing via the NovaSeq 6000 platform using an SP PE250 flow cell and v1.5 Reagent Kit. Raw read pairs were quality-filtered and trimmed using TrimGalore! v0.6.7 (stringency:3; -e:0.2). Long-read libraries, prepared from the same extracted DNA using the Rapid Barcoding Kit SQK-RBK004 (without size selection), were sequenced with a Spot-ON Flow Cell (vR9), MinION sequencer, and MinKNOW v3.1.8 software, with base-calling by a Guppy v2.1.3 high-accuracy mode (all by Oxford Nanopore Technologies plc). Final long-read data sets were trimmed by Filtlong v0.2.1 to select high-quality reads to scaffold the assembly. Default parameters were used for all software unless otherwise specified.

**Fig 1:**
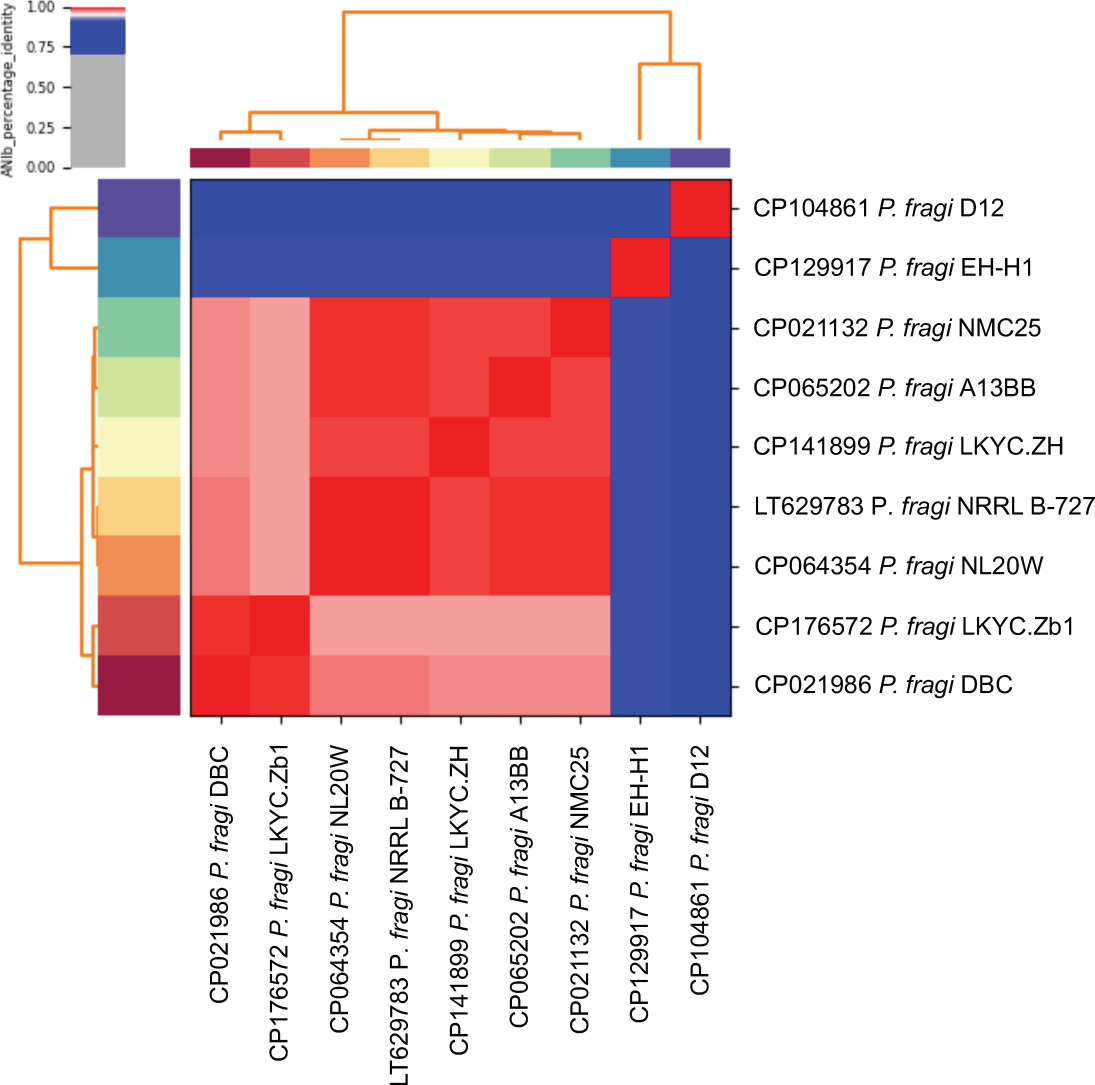
ANI analysis of the LKYC.Zb1 and LKYC.ZH chromosomes and seven complete *P. fragi* chromosomes, as calculated by a BLAST-based method in pyani v0.2.12 (22).

Hybrid assembly, via Trycycler v0.5.5 (LKYC.Zb1) and Unicycler v0.5.0 (LKYC.ZH) (17, 18), yielded a circular chromosome and a single circular plasmid for both isolates, which were submitted to NCBI PGAP v6.9 for annotation (19) (sequencing and assembly data are given in Table 1). pLKYC.Zb1 is a 43,545 bp plasmid encoding a MobF relaxase (20), while the 9,180 bp cryptic plasmid pLKYC.ZH was classified by MobSuite v3.1.9 (21) as a non-mobilizable rep_cluster_1967 replicon. Average nucleotide identities (ANIs) between the LKYC.Zb1 and LKYC.ZH chromosomes and seven complete *P. fragi* chromosomes were calculated by a BLAST-based method in pyani v0.2.12 (22), with the computed relationships displayed in Fig. 1. LKYC.Zb1 and LKYC.ZH were found closest to strains DBC (CP021986) and NMC25 (CP021132), respectively (ANIs > 98.6%).

**TABLE 1:** Sequencing and assembly data for *P. fragi* LKYC.Zb1 and *P. fragi* LKYC.ZH

	LKYC.Zb1		LKYC.ZH	
Sequencing				
Sequencing method	Illumina NovaSeq	Nanopore MinION	Illumina NovaSeq	Nanopore MinION
SRA (raw)	SRR34986797	SRR34986796	SRR34987171	SRR34987170
Filtered reads	1,009,277 (pairs)	108,418	1,648,692 (pairs)	137,383
Mean length	245.9	9,621.9	247.9	9,078.2
N50	250	10,207	250	9,343
Total Mbp	248.2	1,043	408.7	1,247
Assembly				
Assembler used	Trycycler v0.5.5		Unicycler v0.5.0	
Replicon	Chromosome LKYC.Zb1	Plasmid pLKYC.Zb1	Chromosome LKYC.ZH	Plasmid pLKYC.ZH
GenBank accession	CP176572	CP176573	CP141899	CP141900
Topology	Circular	Circular	Circular	Circular
Size	5,205,602 bp	43,545 bp	5,027,131 bp	9,180 bp
G+C %	59.39%	55.4%	59.06%	55.56%
Coverage	293×		248×	
CDs	4,716	48	4,515	13
Pseudogenes	73	0	64	0
rRNA (5S, 16S, 23S)	9, 8, 8	0	9, 8, 8	0
tRNA	73	0	60	0
NCBI loci of genes linked to dye degradation (if present)				
Azoreductase	ACKZH3_15960, ACKZH3_12490		VK847_08680, VK847_12160	
Dyp-type peroxidase	ACKZH3_19820		VK847_04785	
Multi-copper polyphenol oxidoreductase (laccase)	Not present		VK847_07340	
SDR family oxidoreductase (E-value)^*a*^	ACKZH3_15785 (2e-49)		Not present	
	ACKZH3_0478 (1e-16)		VK847_19410 (1e-16)	

^*a*^ Homologs of triphenylmethane reductase (Tmr2) (23) found by NCBI BLASTp (24), with E-values given in brackets.

Both genomes are predicted to encode dye-degrading proteins, including azoreductases (25), a Dyp-type peroxidase (26), and laccase (24). NCBI BLASTp (23) found SDR family oxidoreductases that may be homologs of triphenylmethane reductase (Tmr2) (27). NCBI loci are given in Table 1.

## Data Availability

Sequence data for *Pseudomonas fragi* strains LKYC.Zb1 and LKYC.ZH are available through NCBI under BioProjects PRJNA1198905 and PRJNA1060883, respectively, with corresponding GenBank accession numbers CP176572, CP176573, CP141899, and CP141900, and SRA accession numbers (NovaSeq, MinION reads) SRR34986797, SRR34986796, SRR34987171, and SRR34987170.
